# Non-penetrating traumatic psoas muscle hematoma presenting with gross hematuria: a case report

**DOI:** 10.1186/s12245-021-00345-w

**Published:** 2021-04-07

**Authors:** Lotfolah Abedini, Saadat Mehrabi, Reza Hosseinpour, Mohammad Bagher Jahantab, Vahid Salehi, Mohammad Javad Yavari Barhaghtalab

**Affiliations:** Department of General Surgery, Shahid Beheshti Hospital, Yasuj University of Medical Sciences, Yasuj, Iran

**Keywords:** Non-penetrating, Traumatic, Psoas muscle hematoma, Gross hematuria

## Abstract

Psoas muscle hematoma is defined as a spontaneous or traumatic retroperitoneal collection of blood involving the psoas muscle. Early symptoms of an iliopsoas hematoma include lower abdominal or severe groin pain. Although psoas hematoma is a known complication of coagulopathy, psoas hematoma caused by non-penetrating trauma is the subject of only scattered reports and its significance has not been well described in the literature, so the aim of this study was to report a case of blunt traumatic psoas hematoma with the fracture of vertebral transverse process with the presentation of gross hematuria. A 65-year-old Iranian man slipped backward to the ground, and the patient complaint of gross hematuria and difficulty in walking. There was severe left costo-vertebral angle (CVA) tenderness, and mild groin tenderness, and the lower back area was painful, and he had some pain with the flexion of the vertebral column, and there was tenderness on lumbar spine, but there was no tingling, paresthesia, and weakness in left lower extremity. Hip flexion was 3/5 in the left lower. We used some diagnostic modalities as x-ray radiography, ultrasonography, computed tomography (CT) scan with intravenous (IV) contrast, CT cystography, and intravenous pyelogram (IVP) IVP to differentiate the diagnoses and also find skeletal and other organ injuries associated with this kind of injury. We can conclude that post-traumatic psoas hematoma is a rare condition. The diagnostic modality of choice is CT scan which allows rapid identification and measurement of the hematoma. The lesion usually treated with non-operative conservative management.

## Background

The psoas muscle is among the most important muscles that cover the vertebral column. It is a long fusiform muscle on either side of the vertebral column and the brim of the lesser pelvis. At its distal end, it chains with the iliacus muscle to form the iliopsoas muscle. The function of the psoas muscle is to connect the upper body to the lower body, the outside to the inside, the appendicular to the axial skeleton, and the front to the back, with its fascial relationship. The biomechanical and postural function of the psoas complex muscle is to flex the hip, to adduct the femur, and to externally rotate the hip [1].

Psoas muscle hematoma is defined as a spontaneous or traumatic retroperitoneal collection of blood involving the psoas muscle [2]. Hematomas on the psoas muscle occur secondary to trauma, leaking abdominal aortic aneurysms, iatrogenic etiology during lumbar surgery including endoscopic discectomy, recent biopsy, tumor, use of anticoagulants, or coagulation disorders such as disseminated intravascular coagulation and hemophilia [3, 4]. As a general rule, coagulopathies and trauma are etiologic factors in the younger population while anticoagulant therapy and aortic aneurysm rupture assume prominence in the older age range [4]. The incidence of spontaneous iliopsoas hematoma is 0.1% in general population and 0.6% in elderly patients receiving anticoagulant therapies or affected by coagulopathies [5]. Iliopsoas hematoma (IPH) is associated with a 30 % mortality rate in the intensive care unit (ICU) [2].

Traumatic psoas hematoma is a predictor of severe injury, is closely associated with vertebral column fractures, and accounts for significant blood loss in a minority of patients [4]. Whether incidental diagnosis is frequent, clinical presentation depends on the severity of the hemorrhage, varying from compressive symptoms, such as groin or thigh pain, muscle dysfunction, numbness or paresthesia of the unilateral lower extremity, and occasionally nerve palsy with the femoral nerve involvement and anemia [1, 2]. Ecchymosis in the periumbilical area (Cullen’s sign) or in the flanks (Grey-Turner's sign) may occur [3].

Rapid diagnosis of hematoma is made by the help of sonography and CT imaging. Early signs of hematoma are the diffuse involvement of the muscle and a hyperdense lesion in the muscle in CT images. CT scan is easily and quickly performed and provides useful information in the diagnosis, localization, and finding the volume and spatial extent of the lesion, evidences for an ongoing bleeding or compression on adjacent structures. Furthermore, another main advantage of this imaging modality is its ability to deduce both origin and arterial or venous source of the bleeding [2].

Definitive treatment of iliopsoas hematoma is not well-established. It may be reasonable to consider the following in choosing the method of treatment for hematoma: speed of onset, timing of diagnosis, hematoma volume, cause of hematoma, and degree of neurological impairment. Most hematomas resolve spontaneously without clinical complications [3]. Conservative treatment is reserved for hemophilia patients and those with coagulation disorders [4]. However, in trauma patients, large hematomas or those compressing the femoral nerve with progressive neurological impairment surgical resection is required [3, 6]. Drainage of the hematoma during CT-guided imaging is also useful [3]. Nonetheless, the natural evolution of the injury is spontaneous resolution [6].

Although psoas hematoma is a known complication of coagulopathy, psoas hematoma caused by non-penetrating trauma is the subject of only scattered reports and its significance has not been well described in the literature, so the aim of this study was to report a case of blunt traumatic psoas hematoma with the fracture of vertebral transverse process with the presentation of gross hematuria.

## Case presentation

A 65-year-old Iranian man slipped backward to the ground. He immediately felt left flank and groin pain but was able to continue walking. Approximately 4 h after falling, the pain severely increased, and the patient complaint of gross hematuria and difficulty in walking. He presented to the emergency department of Shahid Beheshti hospital affiliated to Yasuj University of Medical Sciences on the very same day because of gross hematuria. The pain was sharp, non-radiating, and worse with attempting ambulation, and better at rest. He reported no significant medical history, such as hypertension, diabetes mellitus, vascular disease or peripheral neuropathy and clotting or hemorrhagic disorders, and taking antiplatelet or anticoagulant agents. Surgical and genitourinary evaluation revealed no evidence of a hernia or any other disorder.

On physical examination, he was alert, and his vital signs were stable: temperature was 37.60C (afebrile), with a regular pulse of 72 bpm, and blood pressure of 135/70 mmHg. There was skin bruising and ecchymosis in left flank area, but abdomen was soft without tenderness. No pulsatile mass was detected and both femoral pulses were equal. There was no hernia and the scrotal contents were normal, and there was no blood at the urethral meatus. No perineal and scrotal hematoma was seen. The anal tone was good, rectal examination was normal and there was no occult blood, prostate was in normal place on examination and there was no saddle anesthesia. There was severe left CVA tenderness and mild groin tenderness, the lower back area was painful, he had some pain with the flexion of the vertebral column, and there was tenderness on the lumbar spine, but there was no tingling, paresthesia, and weakness in left lower extremity. Hip flexion was 3/5 in the left lower extremity and 5/5 in the right lower extremity. He also had pain on adduction or abduction of the left leg. The straight leg raise (SLR) and FABER tests of left leg were impaired. He was unable to bear weight on the left leg when the patient attempted ambulation. Sensory and motor examinations of the left lower extremity were normal. Ankle jerk reflex (Achilles reflex) was normal in both side, but knee-jerk reflex (patellar reflex) was 1+ in the left side, and 4+ (normal) in the right side.

At the Emergency Department (ED), initial trauma management was done according to the Advanced Trauma Life Support (ATLS) protocol and two IV accesses in the bilateral antecubital fossae were obtained, and urethral catheterization was performed in order to monitor the patient’s urine output, and patient was hydrated with 500 cc of lactated ringer at the initial stage. Focused Assessment with Sonography for Trauma (FAST) scan was done and there was no free fluid in abdomen and pelvic cavity and no solid organ damage.

Hematology revealed normal complete blood count and electrolytes. Coagulation time, prothrombin time (PT) and platelet count did not show any abnormalities. Blood urea nitrogen (BUN) and creatinine (Cr) were 19 and 8 mg/dl retrospectively. Erythrocyte sedimentation rate (ESR) and C-reactive protein (CRP) was 7 mm/h, and 12 mg/dl retrospectively. The result of urinalysis showed 2+ protein, 3+ blood and many red blood cells (RBCs).

**Fig. 1:**
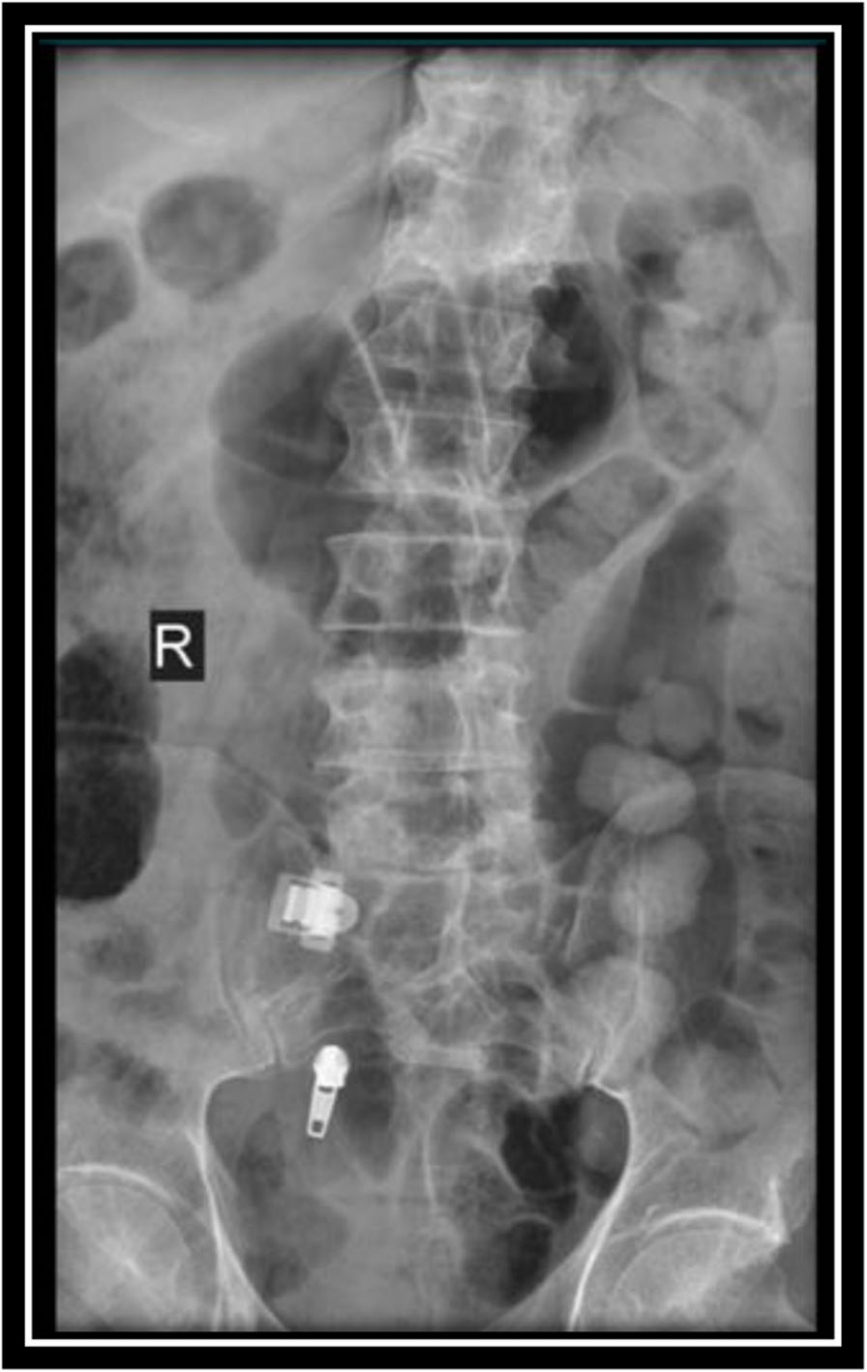
Lumbo-sacral spine X-ray (anterior-posterior view)

**Fig. 2:**
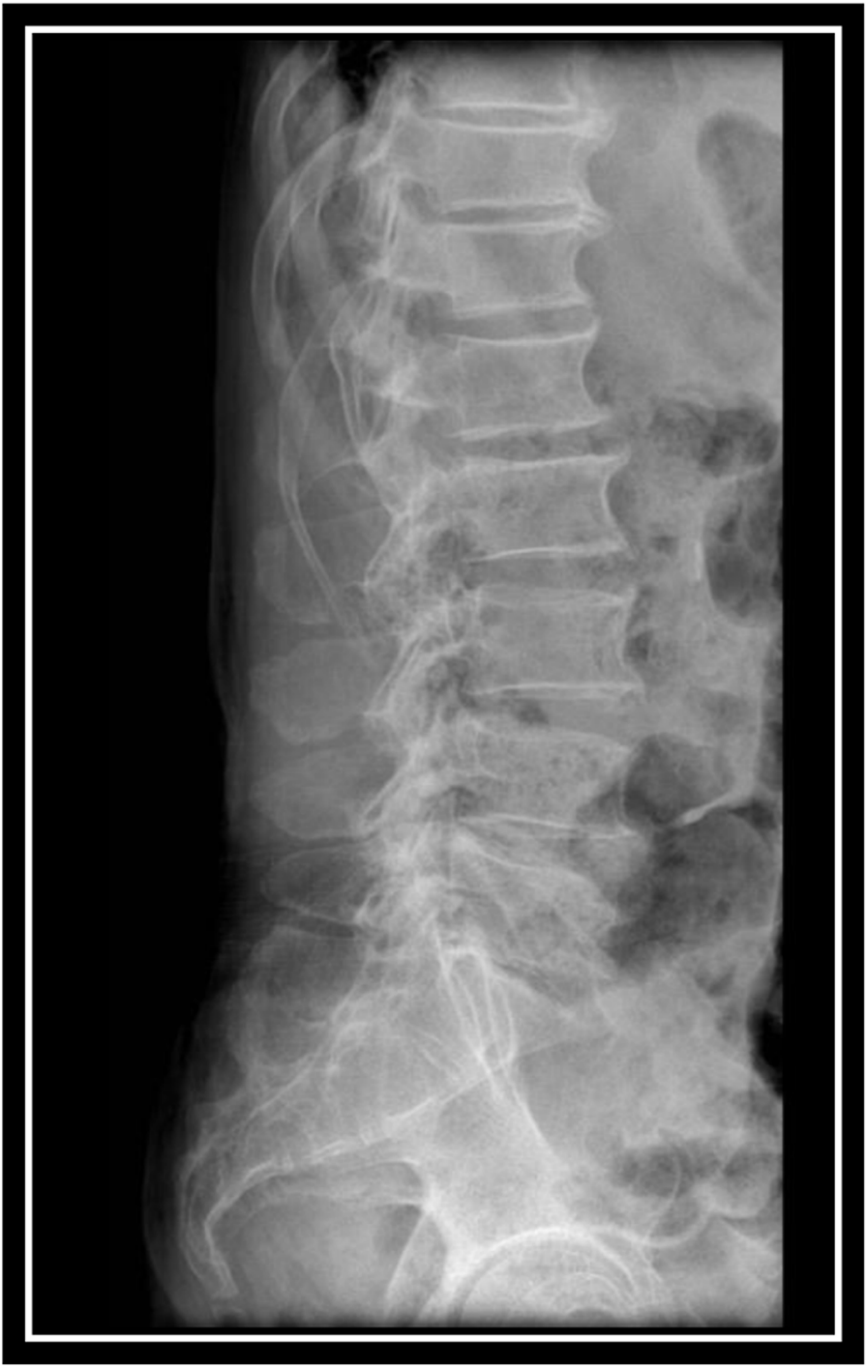
Lumbo-sacral spine X-ray (lateral view)

Radiographs of the pelvis and the lumbo-sacral spine revealed no abnormalities (Figs. 1 and 2). An ultrasound scan obtained on the day of admission revealed a hyperechoic heterogeneous area measuring 48 × 30 mm in the left psoas muscle. Left kidney was enlarged (142 mm) with multiple cortical cyst with the maximum diameter of 46 mm. The intra-abdominal solid organs and adjacent soft tissues were intact, and there was no finding indicative of vascular disorders.

**Fig. 3:**
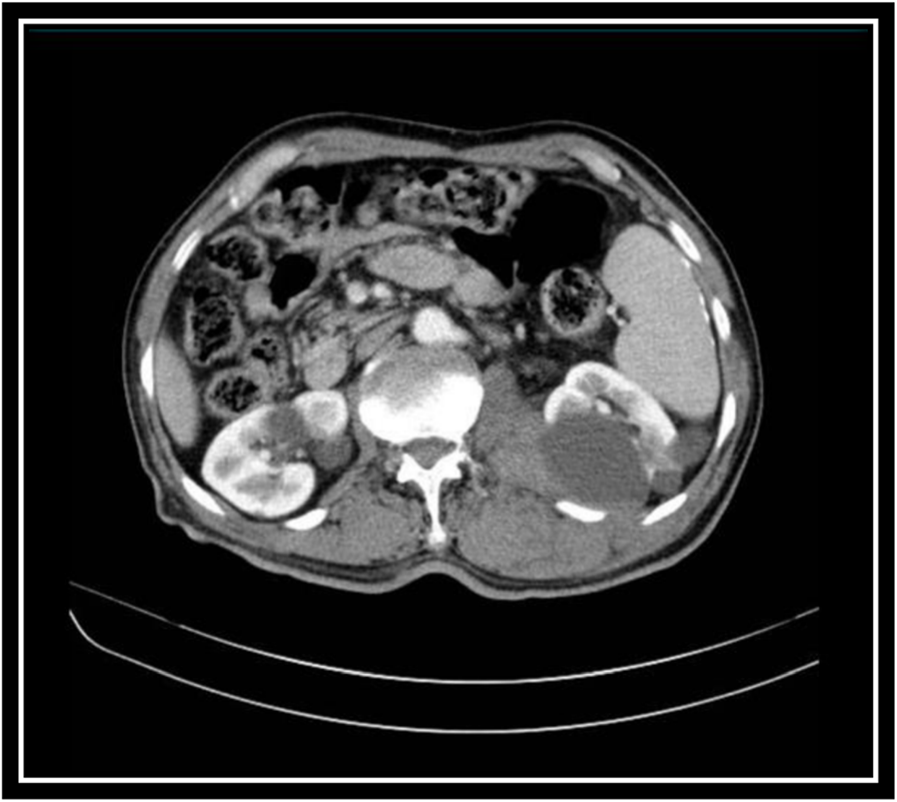
A large left kidney cortical cyst, enlargement of psoas muscle showing hematoma, and fracture of lumbar spine transverse process

**Fig. 4:**
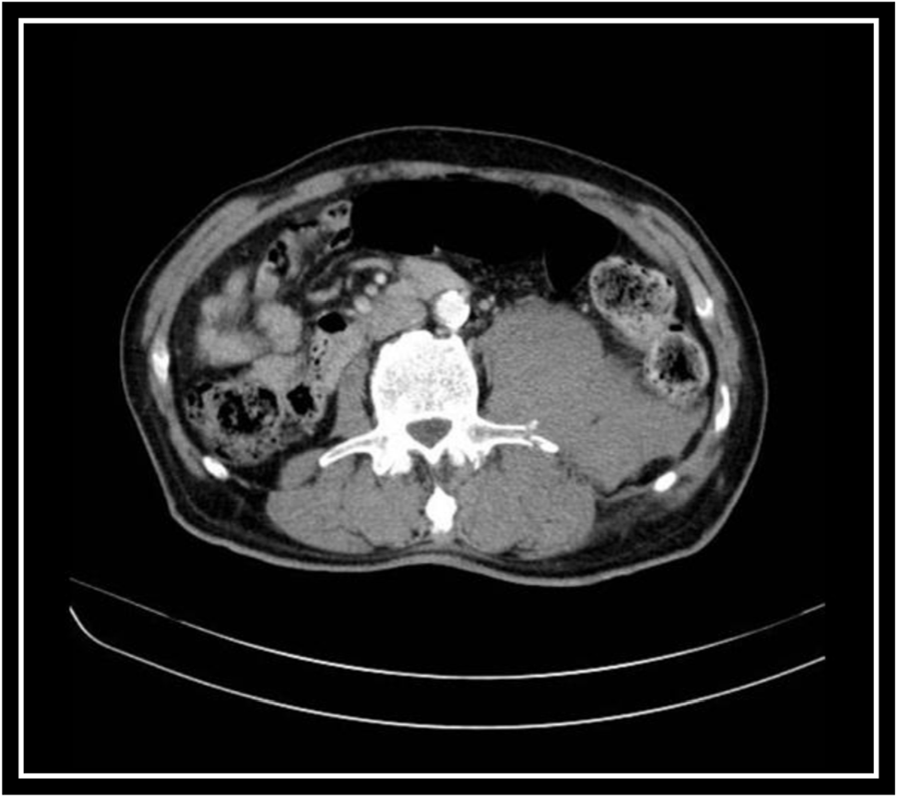
A large left kidney cortical cyst, enlargement of psoas muscle showing hematoma, and fracture of lumbar spine transverse process

The ultrasound scan was compatible with a diagnosis of organizing acute psoas hematoma. A subsequent CT scan was done and revealed multiple large cortical cysts in both kidneys, an enlarged left-sided psoas muscle, and fracture of transverse process of third vertebral spine adjacent to the psoas muscle. CT scan confirmed the presence of psoas hematoma (Figs. 3 and 4).

**Fig. 5:**
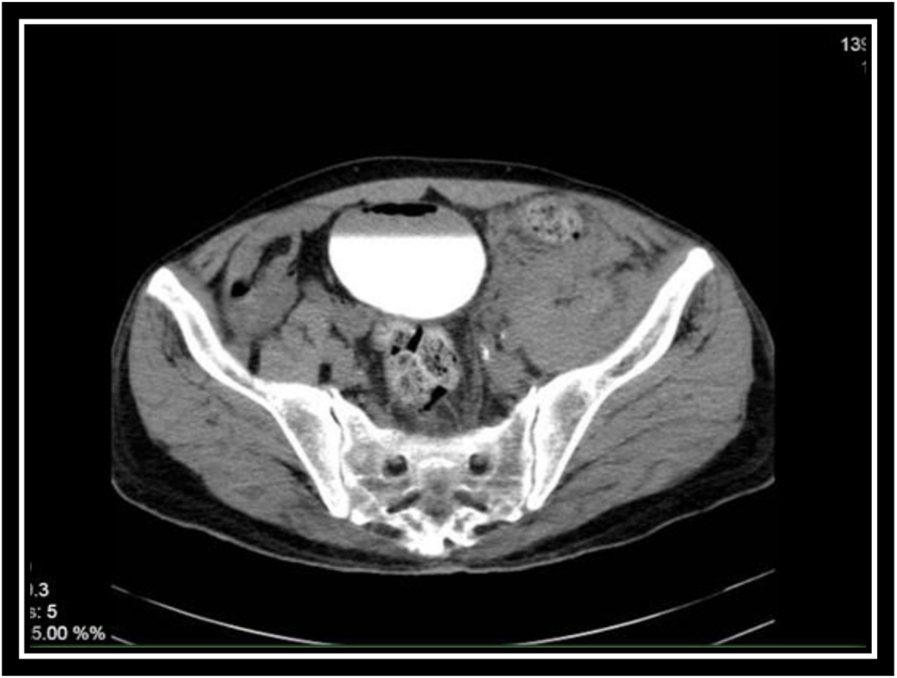
CT cystography (Axial View)

**Fig. 6:**
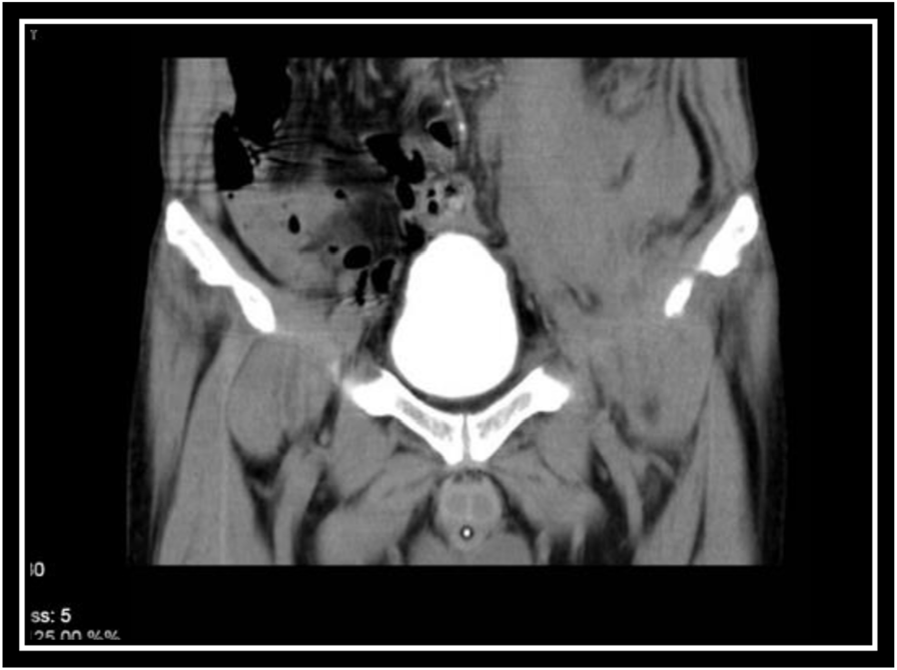
CT cystography (Coronal View)

**Fig. 7:**
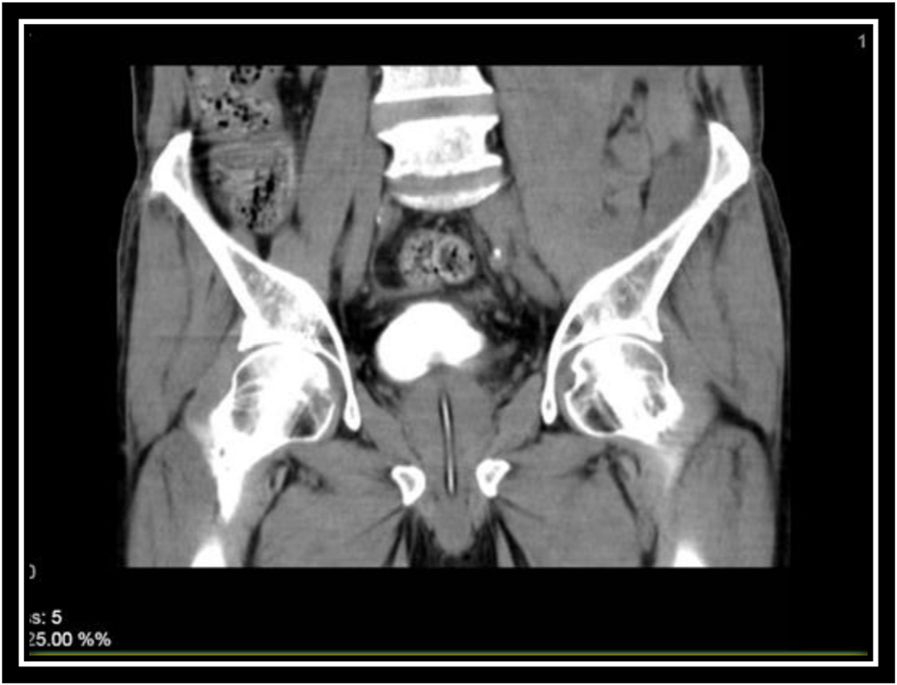
CT cystography (Coronal View)

**Fig. 8:**
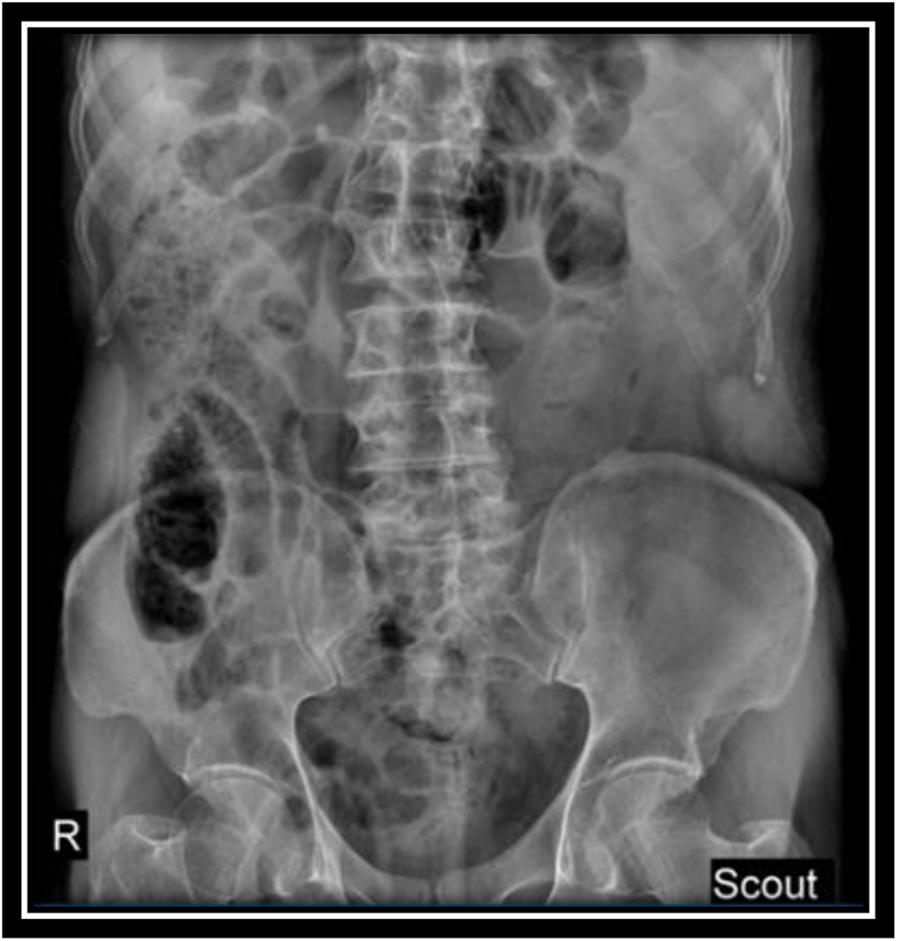
Intravenous pyelography (IVP), before instilling the intravenous iodine contrast (a scout/pilot film)

**Fig. 9:**
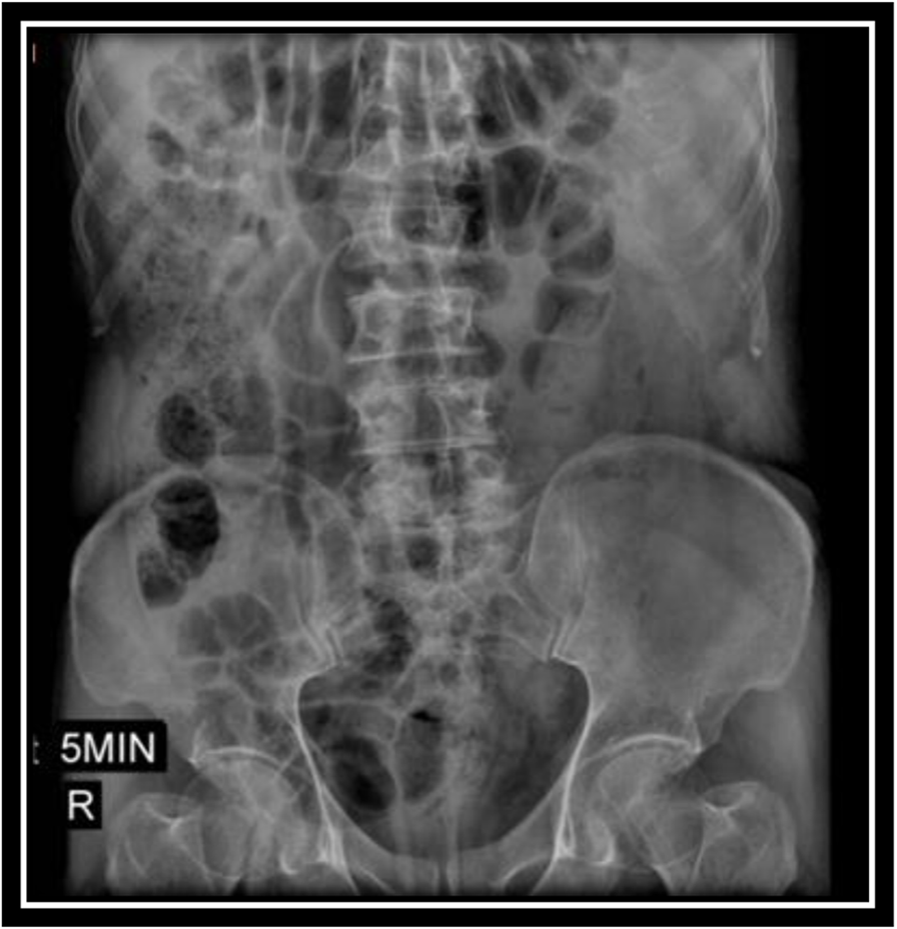
Intravenous pyelography (IVP), 5 minutes after instilling the intravenous iodine contrast

**Fig. 10:**
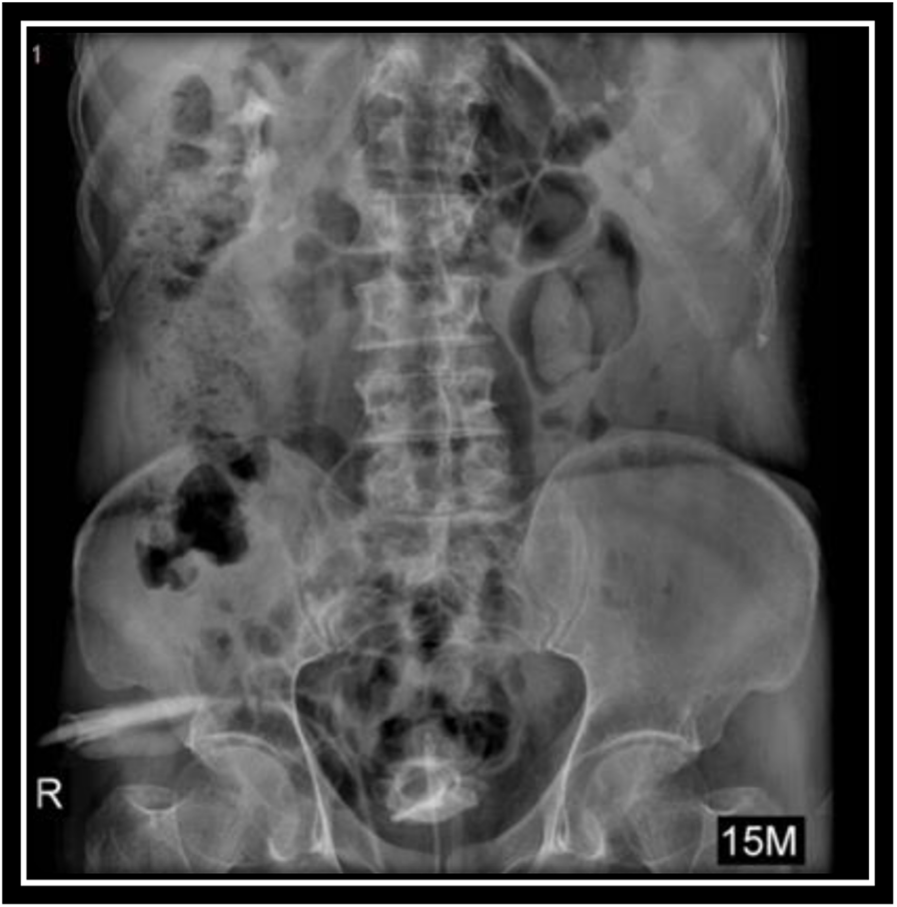
Intravenous pyelography (IVP), 15 minutes after instilling the intravenous iodine contrast

**Fig. 11:**
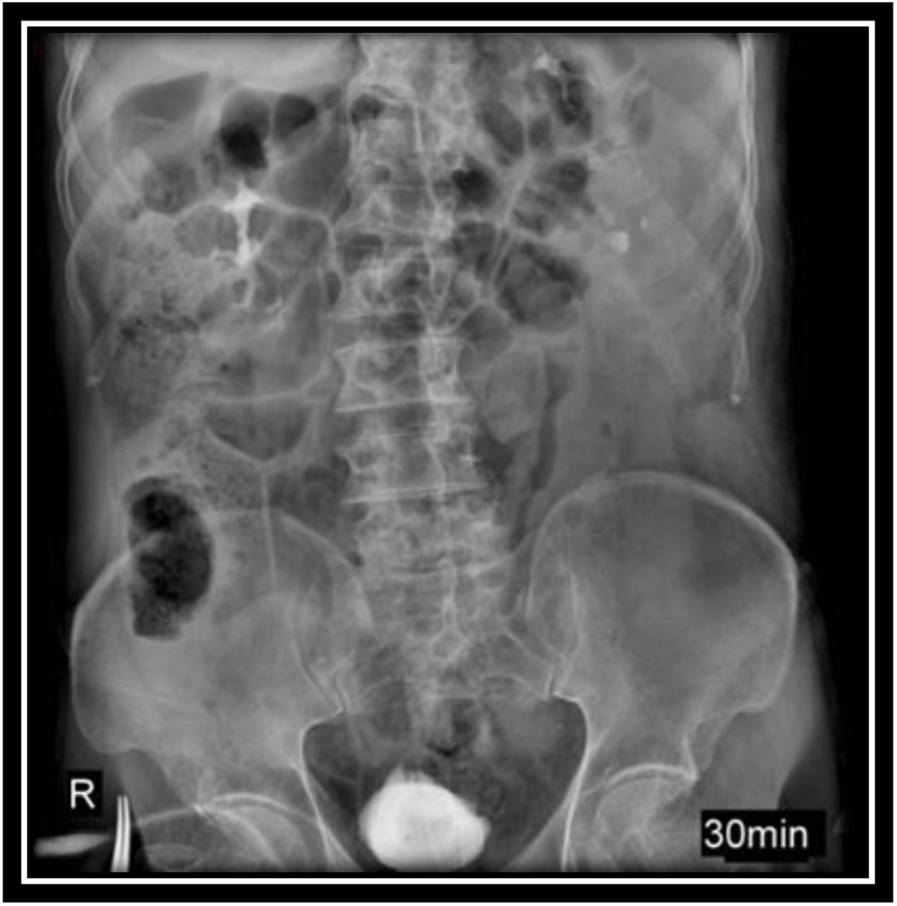
Intravenous pyelography (IVP), 30 minutes after instilling the intravenous iodine contrast

**Fig. 12:**
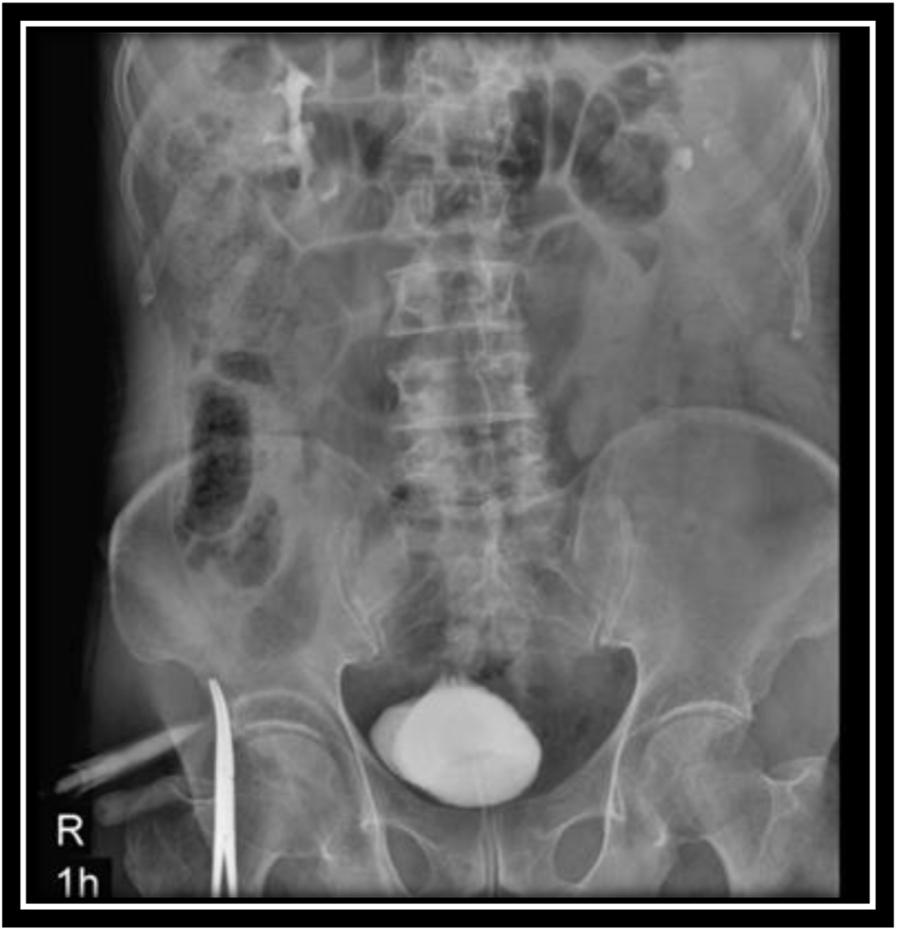
Intravenous pyelography (IVP), 1 hour after instilling the intravenous iodine contrast

**Fig. 13:**
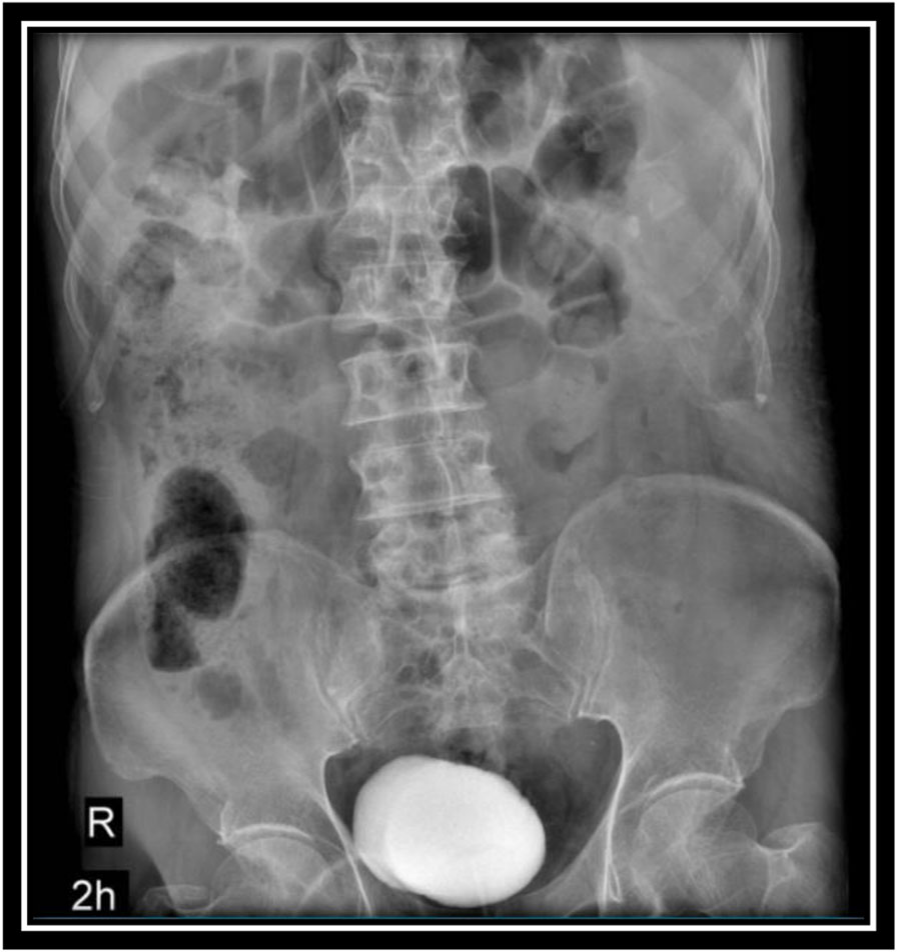
Intravenous pyelography (IVP), 2 hours after instilling the intravenous iodine contrast

**Fig. 14:**
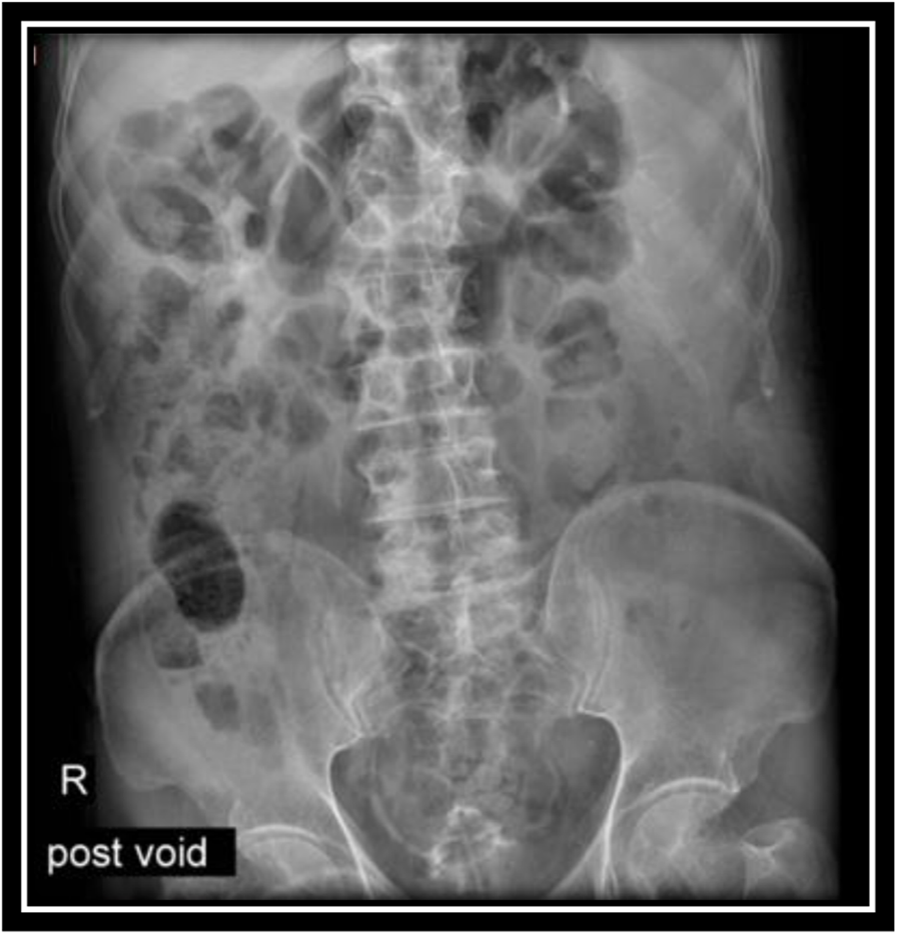
Intravenous pyelography (IVP), Post void image

In order to evaluate bladder for suspected bladder rupture, a CT cystography was done (contrast was instilled retrograde into the patient's bladder, and then the pelvis is imaged with CT) and this revealed no extravasation of contrast or other abnormal pathological findings (Figs. 5, 6, and 7). To evaluate kidneys, ureters, and bladder for gross hematuria, an intravenous pyelogram (IVP) was done next, and it demonstrated no abnormal findings (Figs. 8, 9, 10, 11, 12, 13, and 14).

Hemoglobin levels remained stable for 3 days, thereby excluding active bleeding. Treatment was initialized after confirmation of the diagnosis. Treatment consisted of strict bed rest, conditioning activities, physiotherapy and mobilization, and IV non-steroidal anti-inflammatory drugs (NSAIDs) (Pethidine 50 mg IV every 6 h PRN, and Apotel 1000 mg by IV infusion; it is usually dissolved in 100 ml Sodium Chloride 0.9% Sterile Solution), and they all were started in the hospital, and all of them except the IV NSAIDs were continued on an out-patient basis after discharge 3 days from the injury. A 14-day course of oral analgesic tablets (naproxen sodium; 250 mg every 6 to 8 h) was prescribed. Gradually, the pain eased, and on the first and the second out-patient follow-up visit 7, and 14 days from the discharge the pain was much decreased, and the patient was able to walk without aid. On the 28 days from discharge, the patient was relatively symptom free and returned to his daily routine activities.

## Discussion and conclusion

After systematic history taking and careful clinical examination, it's important to think about relevant differential diagnoses. For this patient who presented with gross hematuria and groin and flank pain after a blunt trauma, the differential diagnoses initially considered were injury to kidney, ureter, bladder, and urethra. We used some diagnostic modalities as x-ray radiography, ultrasonography, CT scan with IV contrast, CT cystography, and IVP to differentiate the diagnoses and also find skeletal and other organ injuries associated with this kind of injury.

This case illustrates an uncommon cause (psoas hematoma and fracture of third lumbar spine transverse process) of a common complaint. The diagnosis of iliopsoas injuries was mostly established after the use of CT scan in this regard. Before the CT scan era, in the past, the diagnosis was difficult and was done through the autopsy or surgery [4].

The pathogenesis of the may be either a tear, a hematoma, or, less frequently, ruptures or synovitis [7]. The presence of iliopsoas injuries is an indication for the severity of force applied to the torso and should lead to a careful assessment for associated injuries in the abdomen, pelvis, or in the vertebral column [4]. Early symptoms of an iliopsoas hematoma include lower abdominal or severe groin pain [8]. Abdominal or flank pain may be present when there is retroperitoneal extension [9]. The lesion may not be palpable in patients examined soon after injury because hematoma takes some time to reach its full size or due to tenderness and guarding [10]. After the trauma, the patients are not able to walk and commonly develop with motor and sensory deficits along the femoral nerve root distribution. The treatment of iliopsoas hematoma remains controversial. The usual treatment for an iliopsoas hematoma is conservative, including bed rest, analgesics, and correction of the presence of a liquefied hematoma. Surgical decompression of the hematoma has been recommended for cases of large iliopsoas hematoma, progressive dysfunction of the femoral nerve, and trauma [8]

The iliopsoas comprises three muscles passing through the pelvis. The origins are on the lumbar vertebrae (psoas major and psoas minor muscles) and the inner pelvis (iliacus muscle) and inserts into the lesser trochanter on the femur. It is the strongest flexor of the hip joint, and with minimal elastic properties, opposes extension of the hip. These muscles are essential in the support of posture and hip flexion. They work together to flex the hip when kicking, bringing the leg out in front of the body or the knee up towards the chest when running [11]. Mechanism of traumatic iliopsoas hematoma is thought to involve excessive iliacus and psoas muscle strain due to abrupt excessive extension of the hip joint [8, 12]. When acting eccentrically at a high angular contraction speed, a muscle develops greater force than produced in a concentric contraction at the same speed. If the muscle stretches by force while acting eccentrically, intramuscular bleeding would occur due to rupture of some its fibers [8].

The iliopsoas muscle is contained in four strong fascial layers, separated by connective tissue or fat, strengthening its intrinsic fascia [9]. The increase in the amount of intramuscular bleeding gradually results in pain through an increase in the intra-compartmental pressure and a local inflammatory reaction, and femoral neuropathy [8, 13].

The association between psoas hematoma and fractures of the transverse processes of the lumbar vertebrae suggest that direct blunt trauma to the back or flank may relate as to cause-and-effect. Transverse process fractures are not usually dangerous, however, a forceful lateral flexion-extension may cause partial avulsion or disruption of the psoas muscle, and leading to intramuscular hemorrhage, hematoma, and associated abdominal, thoracic, or genitourinary injuries [4]. The affected level of vertebral fracture in our study was the third lumbar vertebrae, which is compatible with the other previous studies [4, 14].

Renal cysts could be ruptured and become a source of significant pain. Rupture can occur due to blunt trauma or increased intracyst pressure in the setting of intracyst hemorrhage or infection. Hematuria is seen in up to 84% of patients with cyst rupture, likely resulting from cyst rupture into an adjacent calyx. Less commonly, cysts can rupture through the renal capsule and cause a perirenal urinoma or hematoma [15]. In our case, the reason for gross hematuria seems to be the rupture of the left renal cortical cyst into the adjacent calyx and that's because of the fractures of the transverse processes of the lumbar vertebrae and subsequently psoas muscle hematoma due to a non-penetrating trauma to the left flank area.

The lesion usually disappears by absorption without sequelae [10]. In the absence of progressive neurological findings, if the hematoma is not large, or it is not compressing the surrounding important structures, non-operative conservative treatment is suggested for several reasons. Exploration of the retroperitoneum carries the risk of further bleeding after removal of the clot. There is also the potential for iatrogenic nerve injury and infection [3, 16]. So conservative including rest and ambulation on crutches with no weight bearing on the involved side for a sufficient period led to an uncomplicated resorption of the hematoma [10]. In our patient, the hematoma gradually resolved with no long-term effect on patient's daily activities.

We can conclude that post-traumatic psoas hematoma is a rare condition. The diagnostic modality of choice is CT scan which allows rapid identification and measurement of the hematoma. The lesion usually treated with non-operative conservative management.

## Data Availability

The datasets used and/or analyzed during the current study are available from the corresponding author on reasonable request.

## Abbreviations

CVA: Costo-vertebral angle
IV: Intravenous
CT: Computerized tomography
IVP: Intravenous pyelogram
ICU: Intensive care unit
SLR: Straight leg raise
ATLS: Advanced Trauma Life Support
ED: Emergency Department
FAST: Focused Assessment with Sonography for Trauma
NSAIDs: Non-steroidal anti-inflammatory drugs
PT: Prothrombin time
BUN: Blood urea nitrogen
Cr: Creatinine
ESR: Erythrocyte sedimentation rate
CRP: C-reactive protein
RBCs: Red blood cells

## References

[1] Siccardi MA, Tariq MA, Valle C (2020). Anatomy, Bony Pelvis and Lower Limb, Psoas Major. [Updated 2020 Aug 10]. StatPearls.

[2] Llitjos JF, Daviaud F, Grimaldi D, Legriel S, Georges JL, Guerot E, Bedos JP, Fagon JY, Charpentier J, Mira JP (2016). Ilio-psoas hematoma in the intensive care unit: a multicentric study. Ann Intensive Care..

[3] Seo JG, Yang JC, Kim TW, Park KH (2019). Intramuscular Hematoma on the Psoas Muscle. Korean J Neurotrauma..

[4] Abdulkareem Almadani AA, Afifi IN, Maull KI (2008). Traumatic psoas hematoma. A tell-tale sign of severe injury. Panamerican J Trauma..

[5] Estivill Pallejà X, Domingo P, Fontcuberta J, Félez J (1985). Spontaneous retroperitoneal hemorrhage during oral anticoagulant therapy. Arch Intern Med..

[6] Saad Z, Ahmed B, Mostafa R, Hicham B, Lahcen B (2017). Conservative treatment of a psoas hematoma revealed by a lower limb palsy. Pan Afr Med J..

[7] Mozes M, Papa MZ, Zweig A, Horoszowski H, Adar R (1985). Iliopsoas injury in soccer players. Br J Sports Med..

[8] Qian J, Jing JH, Tian DS, Zhang JS, Chen L (2014). Safety and efficacy of a new procedure for treating traumatic iliopsoas hematoma: a retroperitoneoscopic approach. Surg Endosc..

[9] Choa GPH, Lim CS (2011). Iliopsoas Haematoma: An Uncommon Differential Diagnosis for Groin Pain. Hong Kong J Emerg Med..

[10] Sferopoulos N (2016). Traumatic iliopsoas hematoma: a case report. Orthop Surg Traumatol..

[11] Janse van Rensburg C, Strauss O, Velleman M, Jansen van Rensburg A, Grant C (2015). Iliopsoas haematoma in a rugby player. South Afr J Sports Med.

[12] Kameda T, Fujita M, Takahashi I (2011). Diagnosis of traumatic iliopsoas hematoma using point-of-care ultrasound. Critical Ultrasound J.

[13] Maffulli N, So W, Ahuja A, Chan KM (1996). Iliopsoas haematoma in an adolescent Taekwondo player. Knee Surg, Sports Traumatol, Arthrosc.

[14] Patten RM, Gunberg SR, Brandenburger DK (2000). Frequency and importance of transverse process fractures in the lumbar vertebrae at helical abdominal CT in patients with trauma. Radiology..

[15] Lee J, Darcy M (2011). Renal cysts and urinomas. Semin Intervent Radiol..

[16] Patel A, Calfee R, Thakur N, Eberson C (2008). Non-operative management of femoral neuropathy secondary to a traumatic iliacus haematoma in an adolescent. J Bone Joint Surg Br..

